# Therapeutic Immunization against Glioblastoma

**DOI:** 10.3390/ijms19092540

**Published:** 2018-08-27

**Authors:** Virgil E. J. C. Schijns, Chrystel Pretto, Anna M. Strik, Rianne Gloudemans-Rijkers, Laurent Devillers, Denis Pierre, Jinah Chung, Manisha Dandekar, Jose A. Carrillo, Xiao-Tang Kong, Beverly D. Fu, Frank P. K. Hsu, Florence M. Hofman, Thomas C. Chen, Raphael Zidovetzki, Daniela A. Bota, Apostolos Stathopoulos

**Affiliations:** 1 Epitopoietic Research Corporation ERC-The Netherlands Nistelrooisebaan 3, 5374 RE Schaijk, The Netherlands; a.strik@ercnetherlands.nl (A.M.S.); r.gloudemans@ercnetherlands.nl (R.G.-R.); tstath@hotmail.com (A.S.); 2 ERC-Belgium Gembloux Isnes, Rue Jean Sonet 10, 5031 Isnes Belgium, Belgium; pretto@ercbelgium.eu (C.P.); devillers@ercbelgium.eu (L.D.); dpierre@ercbelgium.eu (D.P.); 3 Cell Biology & Immunology Group, Wageningen University, 6708 PB Wageningen, The Netherlands; 4 Chao Family Comprehensive Cancer Center, 101 The City Drive Bldg. 23, Route 81, Orange, CA 92868, USA; jinahec@uci.edu (J.C.); mdandeka@uci.edu (M.D.); xkong@uci.edu (X.-T.K.); dfu@uci.edu (B.D.F.); fpkhsu@uci.edu (F.P.K.H); dbota@uci.edu (D.A.B.); 5 Department of Neurological Surgery; University of California Irvine, Irvine, CA 92697, USA; Carrilj2@uci.edu; 6 Department of Neurology, University of California Irvine, Irvine, CA 92697, USA; 7 Department of Pathology, Keck School of Medicine, University of Southern California, Los Angeles, CA 90033, USA; hofman@usc.edu; 8 Department of Neurosurgery, Keck School of Medicine, University of Southern California, Los Angeles, CA 90089, USA; tchen68670@gmail.com; 9 Department of Molecular, Cell, and Systems Biology, University of California, Riverside, CA 92521, USA; zidovet@ucr.edu; 10 Department of Neurosurgery, Euroclinics Hospital, 11521 Athens, Greece

**Keywords:** glioma tumor, brain tumor, immunotherapy, therapeutic vaccine, autologous, allogenic

## Abstract

Glioblastoma is the most common form of brain cancer in adults that produces severe damage to the brain leading to a very poor survival prognosis. The standard of care for glioblastoma is usually surgery, as well as radiotherapy followed by systemic temozolomide chemotherapy, resulting in a median survival time of about 12 to 15 months. Despite these therapeutic efforts, the tumor returns in the vast majority of patients. When relapsing, statistics suggest an imminent death dependent on the size of the tumor, the Karnofsky Performance Status, and the tumor localization. Following the standard of care, the administration of Bevacizumab, inhibiting the growth of the tumor vasculature, is an approved medicinal treatment option approved in the United States, but not in the European Union, as well as the recently approved alternating electric fields (AEFs) generator NovoTTF/Optune. However, it is clear that regardless of the current treatment regimens, glioma patients continue to have dismal prognosis and novel treatments are urgently needed. Here, we describe different approaches of recently developed therapeutic glioma brain cancer vaccines, which stimulate the patient’s immune system to recognize tumor-associated antigens (TAA) on cancer cells, aiming to instruct the immune system to eventually attack and destroy the brain tumor cells, with minimal bystander damage to normal brain cells. These distinct immunotherapies may target particular glioma TAAs which are molecularly defined, but they may also target broad patient-derived tumor antigen preparations intentionally evoking a very broad polyclonal antitumor immune stimulation.

## 1. Introduction

Glioblastoma (GBM) is the most common form of brain malignancy in adults. The annual incidence of this disease is about 3–4 cases per 100,000 individuals. The prognosis for late-stage glioblastoma (World Health Organization grade IV astrocytic glioma) is very poor. The median survival time of untreated tumors is only 3 months, with death mostly due to cerebral edema or increased intracranial pressure. Therapeutic interventions involve surgical resection (when safely feasible), followed by radiotherapy (RT), which has been the standard of care for decades. Since 2005, temozolomide (Tmz) chemotherapy has been added to the standard course of radiation [1], resulting in a median survival time of 14.6 months, an increase of about 2 months. However, the vast majority of patients relapse with limited treatment options left [2,3]. If safely feasible, repeat surgery may be considered, but tumor spreading into the brain and spinal cord will hinder adequate surgical resection. Moreover, the cells from the relapsing tumor are often more resistant to chemotherapy. In general, repeated treatment of recurrent tumor lesions may marginally extend overall survival in patients with good performance status [4,5]. The only treatment option left is the angiogenesis inhibitor bevacizumab, a humanized monoclonal antibody targeting vascular endothelial growth factor (VEGF), a molecule which promotes blood vessel growth into the tumor [6]. This antibody inhibits the growth of new blood vessels towards the tumor, but it is only approved in the USA. The latest therapy to be approved for GBM, in both the USA and in Europe, is the alternating electric fields (AEFs) generator NovoTTF/Optune [7], which may extend median overall survival by about 5 to 24 months [8]. A new treatment option for glioma tumors is the use of therapeutic vaccination. The aim of recently developed therapeutic brain cancer vaccines is to stimulate the patient’s immune system to recognize tumor-associated antigens (TAA) on cancer cells, which results in an effective immune response eventually attacking and destroying the brain cancer cells, with minimal bystander damage to normal brain cells.

## 2. Glioma Tumors

Glioma tumors are debilitating and life-threatening brain cancers since they produce severe damage to the brain leading to a poor survival prognosis. Glioma tumors produce a combination of pathologies, which include focal neurologic deficits, resulting from compression and tumor infiltration into the surrounding brain tissue, compromised vascularization, and an increased intracranial pressure. Clinical symptoms include:

- Headaches are prevalent among 30–50% of patients. These headaches are non-specific and indistinguishable from tension headache. Intracranial pressure may increase as a result of tumor growth.
- Seizures may occur among 30–60% of patients. Depending on the tumor location, seizures may be simple partial, complex partial, or generalized.
- Focal neurologic deficits occur among 40–60% of patients. Patients who survive relatively long, may experience increasing cognitive problems, neurologic deficits resulting from radiation necrosis, communicating hydrocephalus, and occasionally cranial neuropathies and polyradiculopathies from leptomeningeal spread.
- Mental status changes are common among 20–40% of patients. With the advent of magnetic resonance imaging (MRI), brain tumors are increasingly diagnosed at an earlier stage and associated with subtle personality changes.

All of these conditions may result in chronic, debilitating symptoms, which negatively affect the patients’ ability to function normally in work or family life and finally lead to a fatal outcome. Hence, there is a significant unmet clinical need for the therapy of malignant glioma, in particular for the late stage of the disease, where patients are faced with dismal prognosis. Advances in neurosurgery, radiation, chemotherapy and concomitant radiochemotherapy during the past decade have provided only small improvements in clinical outcome. The first-line treatment of glioblastoma is usually surgery, both to confirm the diagnosis and to remove as much of the tumor as possible. Radiotherapy followed by adjuvant systemic temozolomide has produced a median survival of about 15 months, and this regimen is now the standard of care for GBM [1,9].

Despite these therapeutic efforts, the tumor returns in the vast majority of patients. When relapsing, statistics suggest an imminent death dependent on the size of the tumor, the Karnofsky Performance Status, and the tumor localization. A scale (ranging from 0 to 3 points) comprised of these three variables distinguishes patients with good (0 point), intermediate (1 to 2 points), and poor (3 points) postoperative survival and indicates that median survival times are respectively 10.8, 4.5, and 1 month, *p* < 0.001 (95% IC) [10]. The median time interval from re-operation after relapse to death for all patients is 7.4 months [10]. At this stage of progression, the patients may be treated with bevacizumab. Bevacizumab is an approved medicinal product in the US, but not in the European Union (EU). However, the response to bevacizumab is transient and short-lived. After 4–6 months, the patients typically develop progressive physical and mental debilitation, and succumb to the disease soon thereafter [11].

Based on the above, it is clear that regardless of current treatment regimens, glioma patients continue to have dismal prognosis and novel treatments are urgently needed.

### 2.1. Therapeutic Glioma Vaccines

The aim of therapeutic brain cancer immunization is to stimulate the patient’s immune system to recognize tumor-associated antigens (TAA) on cancer cells which results in an effective immune response eventually attacking and destroying the brain cancer cells, with minimal bystander damage to normal brain cells. TAA are antigens expressed by tumor cells and not or less by normal healthy cells [12]. When aiming to develop a therapeutic vaccine to treat cancer patients, the prerequisites for the design of an effective cancer vaccine differ clearly from those for the design of a “conventional” prophylactic (often infectious disease) vaccine. First, it should be realized that the cancer patients who will receive the vaccines are immuno-compromised. Secondly, the tumor target antigens are often self-molecules from the patient and are, therefore, poorly immunogenic. Third, tumors develop mechanisms to escape and suppress the immune system. Thus, the design and the choice of immunomodulatory adjuvants for cancer vaccines, both require special attention, and differ relative to those for prophylactic infectious disease vaccines, which are mostly based on antibody responses. By contrast, cancer vaccines in general, need to be designed to generate T cell immune responses to destroy malignant cells, although not always, to be efficient. Nevertheless, a number of promising glioma brain cancer vaccines have been developed recently and will be discussed below.

#### 2.1.1. Survivin-Targeting Vaccines

Survivin is a protein which is upregulated in a variety of human cancers. It is a family member of the inhibitor of apoptosis (IAP) family proteins, which is expressed during embryonic development, but absent in most normal adult cells [13]. Expression of survivin in tumors is associated with an aggressive phenotype [14], with increased resistance to chemotherapy [15]. One prototype product under study is the SVN53-67/M57-KLH peptide vaccine. It is a synthetic peptide vaccine, containing a 15-mer peptide (DLAQMFFCFKELEGW), with C to M alteration at amino acid position 57, derived from the anti-apoptosis protein survivin. The peptide is conjugated with keyhole limpet hemocyanin (KLH), with potential immunopotentiating and antineoplastic activities [16]. KLH may enhance immune recognition and may promote an enhanced response. As SVN53-67 is weakly immunogenic in humans, the M57 amino acid alteration may lead to greater affinity towards HLA-A*0201 and thus an enhanced anti-tumor immune response. Upon subcutaneous administration of SVN53-67/M57-KLH peptide vaccine, the synthetic peptide is able to bind both HMC class I and II molecules. It may, therefore, activate the immune system to mount both a cytotoxic T-lymphocyte (CTL) as well as a T-helper cell response against survivin-expressing cancer cells. This may result in decreased tumor cell proliferation and ultimately tumor cell death. The study is active, but not recruiting in phase II. For detailed information please see ClinicalTrial.gov web site: NCT02455557.

Another viral vaccine approach involving survivin protein is a conditionally replicative oncolytic adenoviral (CRAd) vector that contains the tumor-specific survivin promoter (S) and a fiber protein polylysine modification (pk7), with potential antineoplastic activity. This is a neural stem cell-based virotherapy, which is based on infection of neural stem cells (NSCs) with the gliomatropic oncolytic adenovirus (OV) CRAd-S-pk7 [17]. This oncolytic virus preferentially replicates and destroys glioma tumor cells. This study is recruiting (in phase I study) according to clinical.gov; for detailed information please see ClinicalTrial.gov web site: NCT03072134.

#### 2.1.2. Rindopepimut/CDX-110

The newly Food and Drug Administration (FDA)-approved vaccine-based therapy, rindopepimut/CDX-110, has demonstrated an extension of median survival. However, this vaccine is only applicable to those 30% of GBM patients who are positive for an epidermal growth factor receptor variant EGFRvIII [18]. Unfortunately, in a phase III study there was no significant difference in overall survival for patients with minimal residual disease (MRD): median overall survival was 20.1 months (95% CI 18.5–22.1) in the rindopepimut group versus 20.0 months (18.1–21.9) in the control group (HR 1.01, 95% CI 0.79–1.30; *p* = 0.93) [19].

#### 2.1.3. DCVax Brain

DCVax is an autologous dendritic cell vaccine for newly diagnosed glioblastoma patients [20]. This vaccine showed an excellent safety profile and promising results in an interim result analysis of the latest phase III clinical trial against glioblastoma. In the trial, patients were randomized to receive temozolomide plus DCVax^®^-L (an autologous tumor lysate-pulsed dendritic cell vaccine) or temozolomide and placebo. The median survival of 331 treated patients was 23.1 months from surgery. In comparison, median survival for newly diagnosed glioblastoma patients with the standard of care (surgery, radiation and chemotherapy) is 15–17 months [21]. The trial is blinded and ongoing (NCT00045968).

#### 2.1.4. ICT-107 (a Six Synthetic Antigen Peptide Vaccine)

ICT-107 is also an autologous dendric cell vaccine, which consists of a patient’s own dendritic cells (DCs) loaded with six synthetic peptides from antigens (TAA) associated with glioblastoma tumor cells. The six tumor-associated antigens include: absent in melanoma 2 (AIM-2), melanoma-associated antigen 1 (MAGE-1), tyrosinase-related protein 2 (TRP-2), glycoprotein 100 (gp100), epidermal growth factor receptor 2 (HER-2), and interleukin-13 receptor subunit alpha-2 (IL-13Ra2) [22]. In 124 newly diagnosed GBM patients following surgery and chemoradiation, a randomized, double-blind, placebo-controlled Phase 2 study (NCT01280552) did not show an overall survival benefit [23]. However, the study showed two to three months progression-free survival that was statistically significant when compared with patients treated with DC vaccine that were not pulsed with antigens. In a subsequent randomized, double-blind, placebo-controlled phase 3 clinical trial, in newly diagnosed GBM patients following surgery and chemoradiotherapy (NCT02546102), preliminary analysis showed that four of the targeted antigens were associated with prolonged survival. However, the study was suspended in June 2017 for financial reasons [24].

#### 2.1.5. GLIOVAC/ERC1671

GLIOVAC/ERC1671 is a vaccine based on surgically-resected tumor tissue. The exact active ingredients of gliovac are not defined at the molecular level. Instead the active ingredients are defined as a broad number of tumor-associated antigens, derived ex vivo post-surgery from a glioma tumor as confirmed by a histopathology. Hence, the tumor material isolated by surgery serves as the source of tumor-associated antigens that is required for induction of a pleiotropic anti-tumor immune effector response by the patient’s immune system. This immune response is directed against multiple targets in the non-resected remnants of the glioma tumor bed, that are either not removed by surgical ablation or evolve as new tumor outgrowth from these remnant tumor cells. The rationale, the preclinical and clinical development of this prototype vaccine will be described in more detail below.

### 2.2. The Rationale for a Therapeutic Vaccine Made from Resected Tumor Tissues

In the past researchers developed vaccine preparations by isolating and culturing pure cancer cells from the malignant mass, believing (hoping) they had extracted the essence of the cancer. But in doing so, they omitted connective tissue and other parts of the tumor’s unique biological profile, helping to explain why the majority of cancer vaccines are unable to prevent immune escape of the non-removed tumor cells that evolve post-surgery and often acquire a different antigenic make-up that is not (sufficiently) recognized by the vaccine-induced immune cells. GLIOVAC/ERC1671 was designed to prevent immune escape [25]. GLIOVAC/ERC1671’s active ingredients are from freshly resected, non-cultured glioma tumor cells, aiming to provide a broad set of antigens, which cover as much as possible both the antigenic make-up of patient’s glioma tumor, as well as the antigenic profile of potential newly evolving tumor cells that are likely to appear post-surgery from non-ablated tumor tissue. Therefore, the GLIOVAC/ERC1671 treatment was designed to harbor not only antigens derived from autologous tissue but, in addition, also antigens formulated from allogeneic donated glioma tumor tissue of other patients. These allogeneic antigens further broaden the antigen target profile of in the vaccine. Moreover, the use of allogenic material also triggers a potent anti-‘non-self’, i.e., anti-allogeneic, immune response in the patient. The combination of the autologous with the allogenic antigen approach breaks immunotolerance, by enabling the patient’s immune system to recognize the tumor cells which generally exhibit low immunogenicity. The inclusion of autologous cells allows for a complete personalized treatment for each patient (a single and specific treatment per patient), thereby avoiding the unspecified generic focus of the classical therapeutic immunization approaches. Hence, the allogeneic cells can be regarded as an immunostimulatory therapeutic vaccine ‘adjuvant’ which concomitantly increases the number of cancer-associated tumor antigens (TAA) to be recognized after vaccination by the patient’s immune system.

### 2.3. Pre-Clinical Gliovac/Erc1671 Development

#### 2.3.1. Proof-of-Concept, Composition, Dosing and Timing

Research in rodent (rat) models, designed by the scientific team of Epitopoietic Research Corporation (ERC), provided the proof-of-concept of the vaccine design and gave insight into the critical aspects of the therapeutic anti-tumor intervention strategies, helping to analyze the basic mechanisms of action. Pharmacokinetics and toxicology were then evaluated in mouse studies. The proof-of-concept of the vaccine design, based on the concept that allo-immune reactivity evokes anti-tumor immunity against an autologous tumor, was first observed in two syngeneic glioma tumor rat models. The anti-tumor effect was tested in a therapeutic tumor vaccine setting using two different rat glioma models. The 9L glioma tumor is autologous to Fisher 344 rats and allogeneic to the Sprague–Dawley (SD) rats, while C6 glioma tumor cells are autologous to SD rats and allogeneic to Fisher 344 rats. Therapeutic immunization with a combination of allogeneic cells and autologous lysates induces rejection of malignant autologous gliomas and offered a protective effect against a challenge with autologous tumor cells in both rat tumor models. The results confirmed a protective effect against challenge with autologous tumor cells [26].

Subsequently, the CNS-1 Lewis rat glioma model was used to explore the protective efficacy of various conditions in the vaccine preparation, as well as variations in the dosing and timing schedule. In addition, it was tested whether particular costimulatory agents were able to confer better immunity against CNS-1 tumor development when combined with the allo- and autologous tumor antigen preparation. The results showed that the prototype, consisting of a mixture of allogeneic and autologous glioma cells and their lysates, is able to inhibit CNS-1 glioma growth in the autologous Lewis rats as published in Chapter 6 of the thesis of A. Stathopoulos [25].

Finally, in order to mimic the eventual human vaccine design, therapeutic CNS-1 tumor immunizations were evaluated in the Lewis rat model when combined with the cytokine granulocyte-macrophage colony stimulating factor (GM-CSF), following a low-dose cyclophosphamide (Cy) treatment. The data show that the combination of GM-CSF and Cy with the vaccine was most effective in arresting glioma growth progression as published in Chapter 7 of the thesis of A. Stathopoulos, Vaccine antigen preparation used to evaluate therapeutic immunization when combined with GM-CSF [25]. As a result, this prototype formed the basis for further evaluation in glioma patients in a clinical study.

#### 2.3.2. Pharmacology and Toxicology

As a next step a pharmacokinetic analysis of the human prototype vaccine was performed in 2012 and the results demonstrated that there was no human DNA detected after the vaccine injection when detected by PCR at 1 month after the treatment. Briefly, twenty-one (21) 6-week old, female immunocompromised NOD SCID mice were assigned to two treatment groups of 10 animals each, and one animal remained untreated. Group 1 was administered placebo (together with Cy and GM-CSF), and Group 2 was administered the vaccine (together with Cy and GM-CSF). The evaluation of the study results concluded that injection of the human vaccine substances was well tolerated by all animals. Furthermore, a histological analysis did not reveal any toxicity related to treatment (unpublished data). Histopathological analysis of the organs of each animal used in this study showed no histopathological difference between the placebo and human vaccine treated groups after two separate evaluations, confirming the absence of toxic effects of the human vaccine prototype.

### 2.4. GLIOVAC/ERC1671 Is a Vaccine Based on Surgically-Resected Tumor Tissue

The increased knowledge which had been collected from the animal experiments formed the basis for the composition of the vaccine in patients. For clinical evaluation, the production of the vaccine had to be adapted to adhere to good manufacturing production (GMP) standards for human use, according to the required GMP guidelines.

#### 2.4.1. Background Information on Gliovac

The raw material for the vaccine is glioma tumor tissue obtained from brain surgery, the first step in the current standard treatment. Once resected, the tissue is immediately shipped in a sterile container to the Tumor Tissue Bank–The Netherlands (TTB–NL) and the manufacturing site in which both are situated in The Netherlands. The donated tissue, even though it is tumor tissue and normally discarded, is recorded at the Tumor Tissue Bank–The Netherlands, which is formally audited and approved by The Netherlands Authorities. Tissue release requires negative testing for transmissible infectious diseases, according to the laws of tissue donation. Upon release by the tissue bank and transfer to reception at the manufacturing site, the tissue immediately enters in a sterile production process, which mainly consists of tissue dissociation and cell extraction without a culture step. One part of the cells is stored in a sucrose medium, and one part is lysed by osmotic shock in water. Each donated tissue is manufactured by the same procedure. Quality controls regarding sterility of the product are performed at the end of the procedure, hence there is a theoretical risk that a finished product is not released at the end of the manufacturing process in case of non-conformity. Final release of the finished product is dependent on both the conformity of release of the starting material as well as the final product quality. Eventually, the finished product is irradiated in order to make the tumor cells’ replication incompetent.

**Figure 1:**
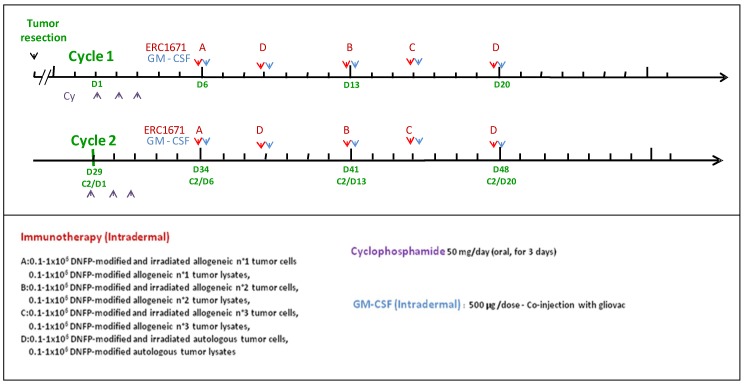
Time schedule of administrations of the treatment. Cyclocphosphamide (CY). ERC1671 doses A, B, C are allogeneic components. ERC1671 doses D are autologous components. Cycle 1 starts on day 1 (D1) with CY administration. ERC1671 is administered on day 6 (D6) with ERC1671 A, day 9 with ERC1671 D, day 13 with ERC1671 B, day 16 with ERC1671 C and day 20 (D20) with ERC1671 D.

GLIOVAC/ERC1671 is a course of immunotherapy, where irradiated/replication-inactivated tumor cells are combined with tumor cell lysate for subcutaneous injection into the glioma patient. The treatment package consists of tumor cells and lysates that are derived from the patient to be treated (autologous component), as well as from three other glioma patient donors (allogeneic component). This package of tumor antigens is administered in a particular treatment schedule (please see Figure 1). Each immunization is given together with GM-CSF (granulocyte-macrophage colony-stimulating factor) to support local immune system priming. In addition, a low dose of cyclophosphamide (Cy) is given a few days before each immunization cycle to deplete immune inhibitory cells in the patient (please see below the treatment details). By using this injection schedule, GLIOVAC/ERC1671 evokes an oligoclonal and partly allo-specific immune induction, based on the application of a broad set of tumor antigens derived from freshly resected glioma tumor tissues from the patient and three unrelated tumor tissue donors.

#### 2.4.2. The Treatment

One treatment cycle is composed of 6 vials of material derived from 3 other patients/donors (allogeneic) and 4 vials of material derived from the patient (autologous) injected intradermally (see Table 1). For each patient the treatment is composed of 3 injections of allogeneic cells and lysates (Gliovac) A, B, C) plus 2 injections of autologous cells and lysate (Gliovac D) given with 3–4 day intervals (Figure 1). Each finished product batch (A or B or C or D) is produced by the same GMP manufacturing process. A full treatment is composed of GLIOVAC/ERC1671 administered with GM-CSF (Leukine^®^) as adjuvant, following a short three-day treatment period of a low-dose of cyclophosphamide (Endoxan^®^).

### 2.5. Clinical Development

In 2015 a report [27] described the use of GLIOVAC/ERC1671 in a clinical setting. It described the immunotherapy’s effect in one recurrent glioblastoma patient who previously had failed second-line standard of care. Although such patients are generally moribund within a few weeks, the GLIOVAC-treated patient survived for 10 months without any other adjuvant therapy [27]. In 2015, Schijns et al. [28] first described the experience with GLIOVAC/ERC1671 in recurrent glioma patients who were treated with Gliovac in an individual hospital exemption protocol. Six-month survival on the GLIOVAC regimen was 100%, and 12-month survival was 40%, providing initial evidence of low toxicity and promising activity of this new therapy with a highly significant overall survival (OS) increase [28], when compared to historic control patients. Please see below for detailed clinical data from the updated cases report [28].

#### 2.5.1. Clinical Data of Individual Compassionate Use Treatments-Cases Report

The GLIOVAC formulation, as deduced from the animal models, has been used for clinical evaluation in individual patients. A total of 10 patients with a Karnofsky performance status (KPS) above 60 that were treated with GLIOVAC/ERC1671 in a compassionate/single program on a named case basis. Median age was 53 (26–62), with 6/10 female patients (see Table 2). The average KPS was 80 (60–100). Out of these 10 patients, all of whom were in terminal stages of disease, 6 had not received bevacizumab (bvz) during their disease treatment (before, during or after Gliovac treatment). Out of the 4 patients that had received bvz, 2 had bvz treatment until GBM progression, and stopped the bvz prior to surgery and the start of GLIOVAC/ERC1671. One patient had received bvz until disease progression, followed by surgery and 4 cycles of GLIOVAC/ERC1671, and was continued on bvz after the 4 cycles of GLIOVAC/ERC1671 were completed, due to concerns of possible disease progression. One patient received bvz until disease progression and continued on both GLIOVAC/ERC1671 and bvz for an additional 2 months. No significant side effects potentially attributable to the combination were witnessed. To properly assess the results from this study, patients have to be compared to the outcomes of currently used treatment regimens for recurrent GBM patients, which are as follows.

The expected OS for those GBM patients with a good KPS (more than 70), after failing radiation and Tmz, and without access to bvz (as is the case with many patients in the EU), is 5–8 months, and their 6-month progression-free survival (PFS) is around 30% [29]. For patients that have already failed bvz, predicted OS is reported at 4–5.8 months, and 6-month PFS rate is from 4.4% to 16%, depending on the study [30,31].

**Figure 2:**
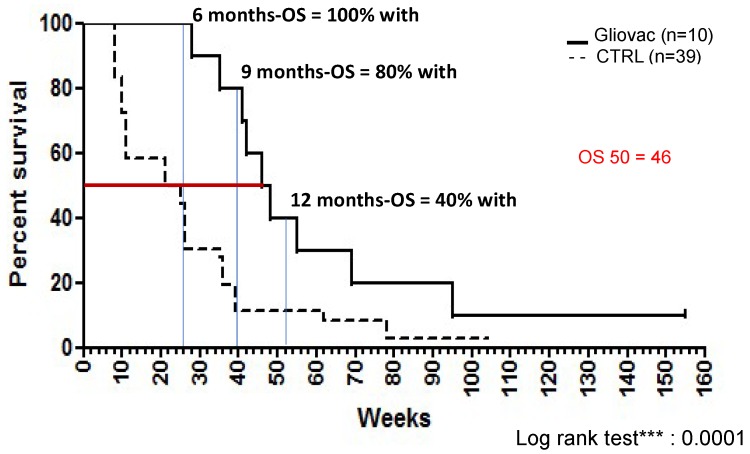
Results obtained from patients treated under compassionate/single-name program. Although data need to be confirmed in a stringent clinical trial, results show a highly significant (log rank test, *** *p*-value = 0.0001) increase of the overall survival (OS) of late stage relapsing patients when treated with Gliovac when compared to historic control patients (study published by Barker et al., 1998 [32]).

In comparison, our dataset of recurrent GBM patients treated with Gliovac shows the following: 6-month OS is 100%, 12-month OS is 40%, and median OS is 46 weeks (10.5 months). Historic controls (data from [32]) have 6-month OS of 33% and median OS of 23 weeks (5.3 months) (Figure 2). Thus, this dataset reveals a striking improvement over current clinical practice. As shown in Figure 2, the results showed a highly significant (log rank test, *p* < 0.0001) increase in the OS of patients when treated with Gliovac. Hence, these results are supportive of the benefit of the combined treatment schedule, administering an immunization package of allogeneic and autologous cells and lysates in repeated cycles of treatment, with minimal toxicity.

#### 2.5.2. Clinical Study Data from a Phase II Food and Drug Administration (FDA) Trial

To further investigate and validate safety and effectiveness of GLIOVAC/ERC1671, an FDA-approved double-blinded, placebo-controlled phase 2, clinical study (NCT01903330), was initiated at the University of California, Irvine. This phase II clinical study, entitled “*ERC1671/GM-CSF/Cyclophosphamide for the Treatment of Glioblastoma Multiforme*” (NCT01903330), is designed to show the anti-tumor efficacy of Gliovac plus GM-CSF plus Cy with bevacizumab (bvz), as compared to placebo injection (instead of Gliovac/GM-CSF) plus placebo pill (instead of Cy) with bvz, in patients with recurrent grade IV malignant gliomas, including GBM. As mentioned before, both treatment arms include bvz. Therefore, this study aims to further validate superior activity of GLIOVAC/ERC1671 as compared to bvz alone. GLIOVAC/ERC1671 data from the compassionate use treatment, without bvz, already shows beneficial 6-, 9- and 12-month OS data in comparison to published bvz monotherapy studies (Table 3) from Taal et al., 2014 [33], Field et al., 2015 [34], Heiland et al., 2016 [35]. Currently, the clinical phase 2 study has started and is recruiting.

## 3. Discussion

Therapeutic immunization against brain cancer, aiming to stimulate the patient’s immune system to (better) recognize and destroy tumor-specific antigens on malignant brain cells would provide a formidable new treatment option for the brain cancer patients for whom little new treatment progress has been made in decades. As described above, several encouraging new vaccine candidates have recently been developed, which showed an effective immune response, eventually attacking and destroying the brain cancer cells with clinically meaningful efficacy and an acceptable safety profile, with minimal bystander damage to normal brain cells.

For both the DCVax treatment and the Gliovac therapy, the tumor target antigens are not defined and characterized at the molecular level (and do not need to be). As a result, both approaches induce a strong and broad polyclonal immune response to multiple tumor antigens present in the antigen preparation, thereby reducing the risk of tumor immune escape following the loss of particular TAAs.

During its evolution, from drawing table to a prototype vaccine for human application, the GLIOVAC product was composed and tested in preclinical studies to harbor a number of characteristics for optimal immune induction. The prototype of GLIOVAC/ERC1671 was designed to contain as much as possible of the “original” tumor cells. Indeed, the isolated glioma tumor cells are not cultured, but directly filtered and conditioned from the surgically resected tumor tissue. This product aspect is particularly important, because in ex vivo tumor cultures only a percentage (about 20%) of the tumor cells survive the switch towards a culture medium environment. Hence, a culture procedure reduces considerably the mutation variety and quantity of TAAs that is normally present in freshly isolated tumor cells. So, avoidance of an in vitro cell culture step maintains the broadness of tumor target antigens in the final vaccine preparation and, hence, the broadness of induced immune power against the target tumor.

Furthermore, the Gliovac treatment not only includes autologous TAAs from the patient, but also includes TAAs from three allogenic glioma tumor donors. These allogenic antigens evoke an immunological phenomenon that is comparable to a “graft rejection” due to the presentation of allogeneic cells which are HLA-incompatible. The allogenic vaccines are prepared from donated tumor tissue in a process identical to the vaccine preparation for the autologous antigens. Upon recognition of and “immune rejection” of the injected allogeneic tumor antigen preparation, the immune system of the patient will develop an immune response recognizing cancer cell associated-proteins, including the so-called tumor associated antigens (TAA) overlapping with those from the patient, and consequently reject the patient’s own tumor.

Hence, allorecognition and allo immune-induction is a key ingredient in the mechanism responsible for allograft immune rejection (reviewed in Fabre, 2001; also in Gervais, 2009) [36,37]. It is well known that unprimed T lymphocytes from one individual react with unusual strength against HLA antigens of other members of the same species, a phenomenon called “allo-agression”. This process is based on the direct T-cell allorecognition. It reflects the capacity of T lymphocytes to recognize intact allogeneic HLA molecules on the surface of foreign cells. It is a powerful mechanism of T-cell activation, since about 1–10% of an individual’s T lymphocytes will respond to the foreign HLA antigens of another individual. By comparison, the frequencies of T-cell precursors for “normal” environmental antigen (e.g., a virus protein) are of the order of only 1/10,000 or 1/100,000.

The injection of autologous and allogeneic glioma tumor antigens has the advantage that it exposes the patient’s immune system to a larger variety of tumor antigens, which increases the chances to trigger essential immune effector cell populations. In addition, donated allogenic tumors warrant the availability of a critical quantity of active substance. In contrast to immunizations based on cell lines only, the autologous/allogeneic biopsy-based immunizations depends on the size of the tumor isolated during surgery from the patient and the donor. However, by using allogeneic donor tumor tissue, a theoretical limitation in active substance is partially circumvented, since a large part of the finished product can be obtained from a tumor tissue bank.

Both CD8 and CD4-positive T-cells are implicated in the allorecognition phenomenon. CD4 T-cells activated by direct recognition of HLA class II molecules during immunization may act as providers of T-helper activity, triggering and sustaining a TAA-specific immune response against the patient’s own tumor cells. In organ transplant rejection, this powerful activation of T-helper cells is responsible for an early antibody response against the transplant. This is particularly important in tumor therapy as it could theoretically bypass the need for presentation of TAA within self HLA class II molecules to activate T-helper cells and could induce a powerful cellular and humoral immune reaction against tumor cells displaying TAA within HLA class I antigens.

In keeping with the above, it is worth mentioning that CD4 T-cell responses are not only necessary, but may also be sufficient for allograft destruction. Allogeneic responses have the potential to generate a milieu rich in cytokines sustaining both an innate immune reaction and promoting a T-cell response by providing T-cell costimulatory ligands. The exact number of CD4+ T cells, or other relevant immune cells, required for effective immunotherapy is currently subject of various clinical studies and remains to be determined. This may be especially relevant for immunotherapies in patients with depressed immune cell numbers as a result of chemotherapy. The use of Gliovac as an upfront treatment before cytotoxic therapy would likely circumvent this problem. Collectively, it is apparent that allorecognition can be used as a potent mechanism to stimulate a glioma TAA-specific immune reaction against a patient’s tumor cells.

It should be kept in mind that immune cells triggered in the periphery by therapeutic immunization are undoubtedly able to cross the blood–brain barrier (BBB), which is “disrupted” in gliomas. Malignant gliomas actively degrade previously intact endothelial tight junctions of the BBB by secreting soluble factors, eventually leading to BBB disruption within invaded brain tissue as confirmed in neuroradiological examination [29,30].

Unfortunately, the evaluation of the patient using classical radiological imaging techniques becomes (more) complex. When using immunotherapy it is difficult to discriminate between progression of tumor growth versus pseudoprogression resulting from immune cell infiltrates. Therefore, the advent of immunotherapies implies the use of other evaluation techniques such as clinical parameters and overall survival of the patient.

## 4. Conclusions

The composition and regimen of GLIOVAC/ERC1671 appear to offer unique and compelling advantages. In comparison to historical controls, GLIOVAC therapy in individual late-stage relapsing patients resulted in significantly increased OS-6 month (100%) and greater median OS (46 weeks compared to 23 weeks). Moreover, current clinical data show that the product is safe, with no severe adverse effects (AE) observed. Related toxicities were mainly limited to low grade headaches and local skin reactions.

Moreover, after GBM recurrence following standard care treatments, about 10% of the case-reported patients treated with GLIOVAC/ERC1671 showed a total recovery and survived longer than 3 years in the compassionate/single-name program. Importantly, spontaneous remissions have never been observed in the relapsed GBM patients showing tumor progression. Despite the limited number of patients, the overt remission of patients, to our knowledge, is the best example of the product’s efficacy.

The primary results in clinical evaluation based on combined allogenic/autologous antigen preparation, which has been developed in an animal model, are supportive of the product’s rationale. The meaningful efficacy against relapsing high-grade glioma combined with very low toxicity of GLIOVAC/ERC1671, and promising clinical data of other vaccine candidates, indicate that therapeutic immunization against glioblastoma comes within reach for patients suffering recurrent refractory disease with no therapeutic option or choice left.

## Figures and Tables

**Table 1:** Composition of one cycle of treatment.

Dose Sequence	Composition
[Gliovac] A	Vial 1: 1 × 10^5–6^ allogeneic n°1 glioma tumor cells
	Vial 2: 1 × 10^5–6^ allogeneic n°1 glioma tumor lysates
[Gliovac] D	Vial 3: 1 × 10^5–6^ autologous glioma tumor cells
	Vial 4: 1 × 10^5–6^ autologous glioma tumor lysates
[Gliovac] B	Vial 5: 1 × 10^5–6^ allogeneic n°2 glioma tumor cells
	Vial 6: 1 × 10^5–6^ allogeneic n°2 glioma tumor lysates
[Gliovac] C	Vial 7: 1 × 10^5–6^ allogeneic n°3 glioma tumor cells
	Vial 8: 1 × 10^5–6^ allogeneic n°3 glioma tumor lysates
[Gliovac] D	Vial 9: 1 × 10^5–6^ autologous glioma tumor cells
	Vial 10: 1 × 10^5–6^ autologous glioma tumor lysates

**Table 2:** Compassionate/single-name patient Characteristics.

Patient Anonymization Code	Clinical Site	Age at Diagnosis	Sex	KPS	OS (weeks)
ERC-B-G2012-54-005	CSL	58	F	80/100	28
ERC-B-G2012-50-011	CSL	62	M	80/100	41
ERC-UCI-40-JS-04031986	UCI	44	M	80/100	42
ERC-B-G2012-51-017	CSL	62	F	70/100	46
ERC-L-G2012-65-019	VIL	49	F	70/100	35
ERC-G-G2012-63-020	UKS	50	M	80/100	69
ERC-B-G2013-58-023	CSL	55	F	80/100	48
ERC-CO-G2014-87-033	FIRE	28	M	100/100	115
ERC-CO-G2014-88-042	FIRE	26	F	95/100	95
ERC-ZA-G2015-62-048	GVI	53	F	70/100	55

KPS is Karnofsky performance status; OS is overal survival.

**Table 3:** OS data of gliovac in comparison to bevacizumab studies. Published Bevacizumab monotherapy studies from: Taal et al., 2014 [33], Field et al., 2015 [34], Heiland et al., 2016 [35].

		6 mo OS	9 mo OS	12 mo OS
Study	Treatment	All Pts (%)	All Pts (%)	All Pts (%)
ERC 1671	ERC 1671	100	80	40
Taal (BELOB)	BEV Monotherapy	62	45	26
Field (CABARET)		61	39	24
Heiland (Freiburg, Germany)		18	12	10

## Abbreviations

Bvz	bevacizumab
Cy	cyclophosphamide
Dc	dendritic cell
ERC	Epitopoietic Research Corporation
GM-CSF	granulocyte-macrophage colony stimulating factor
TAA	tumor-associated antigen
Tmz	temozolomide
